# A clinical evaluation of botulinum toxin-A injections in the temporomandibular disorder treatment

**DOI:** 10.1186/s40902-016-0051-7

**Published:** 2016-01-28

**Authors:** Hyun-Suk Kim, Pil-Young Yun, Young-Kyun Kim

**Affiliations:** 1 Department of Oral and Maxillofacial Surgery, Section of Dentistry, Seoul National University Bundang Hospital, 300 Gumi-dong, Bundang-gu, Seongnam, Gyunggi-do South Korea; 2 Department of Dentistry and Dental Research Institute, School of Dentistry, Seoul National University, Seoul, South Korea

**Keywords:** Botulinum toxin type A, Temporomandibular disorder, Research diagnostic criteria for temporomandibular disorder

## Abstract

**Background:**

This study clinically evaluated the effect of botulinum toxin type A (BTX-A) in the temporomandibular disorder (TMD) treatment using Research Diagnostic Criteria for Temporomandibular Disorders (RDC/TMD).

**Methods:**

A total of 21 TMD patients were recruited to be treated with BTX-A injections on the bilateral masseter and temporalis muscles and were followed up by an oral and maxillofacial surgeon highly experienced in the TMD treatment. For each patient, diagnostic data gathering were conducted according to the RDC/TMD. Characteristic pain intensity, disability points, chronic pain grade, depression index, and grade of nonspecific physical symptoms were evaluated. Wilcoxon signed-rank test was applied for statistical analysis.

**Results:**

The results showed that more than half of the participants (85.7 %) had parafunctional oral habits such as bruxism or clenching. In comparison between pre- and post-treatment results, graded pain score, characteristic pain intensity, disability points, chronic pain grade, and grade of nonspecific physical symptoms showed statistically significant differences after the BTX-A injection therapy (*p* < 0.05). Most patients experienced collective decrease in clinical manifestations of TMD including pain relief and improved masticatory functions after the treatment.

**Conclusions:**

Within the limitation of our study, BTX-A injections in masticatory musculatures of TMD patients could be considered as a useful option for controlling complex TMD and helping its associated symptoms.

## Background

Temporomandibular disorders (TMD), musculoskeletal disorders of the masticatory system, are common clinical labels for pain in the orofacial area. Causes of TMD symptoms are often complex and idiopathic, and problems involve extra-articular and intra-articular pathologic conditions or a combination of the both. TMD patients manifest various symptoms including myofascial tenderness and pain, headache, joint noises, trismus, and even tinnitus. In symptomatic muscle areas, a number of trigger points could be present, and palpating these musculatures generally initiates radiating discomforts along with cascading pains on multiple muscle and neuronal tracks. Depending on contributing factors, these symptoms may be transient or perpetuating [1].

Successful TMD treatment starts from correctly differentiating the origin of symptoms. Since myofascial pains and mouth opening limitation are the most frequent symptoms in masticatory muscle disorders, directing treatments at the muscular components of TMD could yield therapeutic gains. Therefore, noninvasive conservative treatments such as counseling, soft diet, behavior medicine, physiotherapy, oral appliances, pharmacotherapy, and botulinum toxin injections are reported to be effective as first-line therapies for extra-articular pathologic conditions [2].

Intra-articular pathologic conditions such as internal derangement and osteoarthritis could also benefit from the reversible interventions used to treat myofascial pains. Acute patients, however, may require intra-articular injection of lidocaine, hyaluronic acid, or even corticosteroid [3]. When the symptoms and pains surpass the effectiveness of these techniques, surgical approaches such as arthrocentesis, arthroscopy, and TMJ open surgeries such as arthroplasty may be performed to treat anatomical pathology.

The purpose of this study was to clinically evaluate the effect of botulinum toxin injection in treating TMD arising from both intra-articular and extra-articular pathologies based on Research Diagnostic Criteria for Temporomandibular Disorders (RDC/TMD) which provides a comprehensive assessment.

## Methods

### Subjects

A total of 21 TMD patients (17 females, 4 males; 4.25:1) were recruited from Feb. 2007 to Sept. 2013 to be treated with botulinum toxin type A (BTX-A) injection. The participants had chief complaints of myofascial pain, trismus, and/or TMJ sounds. The mean age of patients was 37.0 ± 15.1 years (ranged from 15.2 to 69.0 years). A detailed sex and age distribution is shown in Table 1.

**Table 1:** Sex and age distribution

Age	Sex		Total
	Male	Female	
15 to 19	1	2	3
20 to 29		3	3
30 to 39	3	5	8
40 to 49		3	3
50 to 59		2	2
Over 60		2	2
Total	4	17	21

The subjects filled out the Korean-translated version of RDC/TMD Axis II questionnaires before and after the end of the treatment [4]. The patients who did not complete the questionnaires were removed from the study group. The medical charts were reviewed by a trained oral and maxillofacial surgeon along with the clinical record, radiographic examination, and bone scan. RDC/TMD Axis I criteria were used to diagnose the TMDs, and they were further classified under the TMD subtypes proposed by the Japanese Society for the Temporomandibular Joint (JSTMJ) in 2001: type I, masticatory muscle disorder; type II, capsule-ligament disorder; type III, disc disorder; type IV, degenerative joint diseases, osteoarthritis, and osteoarthrosis; and type V, cases not included in types I–IV [5]. The main diagnosis category was selected when multiple diagnoses were given. Inclusion criteria were as follows: (1) patients who received BTX-A injection therapy during the study period, (2) patients with complete medical records, and (3) patients with TMD/RDC follow-ups.

Before the BTX-A treatment, all patients had undergone noninvasive conservative therapies including counseling, pharmacotherapy, behavior medicine, and physical therapies. As for first-line pharmacotherapy, various medications such as NSAIDs, corticosteroids, analgesics, tricyclic antidepressants, and muscle relaxant were prescribed depending on the pathologic conditions of the patients. Physical therapies were performed in forms of low-level laser therapy (LLLT), ethyl chloride spray and stretch technique, jaw stretching exercise, and stabilization splints.

This research was approved by the Institutional Review Boards of the SNUBH (IRB #: B-1305-204-110), and informed consent was obtained from all participants prior to initiating the study.

### BTX-A injection technique

BTX-A powders (Dysport®, Ipsen Ltd, Slough, UK) were kept frozen in sterile vials until each use. Preparation of the BTX-A solution was done under the manufacturer’s guidelines. The solution was prepared by adding 0.9 % normal saline without a preservative to the powders until 2 ml of final dilution. Injection sites were wiped with 70 % ethanol swab, and dry sterile gauze for skin preparation and aspirations were performed before each injection. Calibrated 1.0-ml tuberculin syringes with 26 gauge needles were used for the injection. The prepared solution was used within an hour for its maximum potency.

The patients received a total of 500 U of Dysport (BTX-A) injections into the bilateral masseter and temporalis muscles with the injection ratio of 3 to 2, respectively. Before injections, patients were asked to clench their jaws to make the injection sites more prominent. Using multiple injection technique, each masseter and temporalis muscle had three injection sites, with diffusion of approximately 1 cm apart from each sites. The treated patients were followed up by a dentist highly experienced in the TMD treatment.

### RDC/TMD Axis II assessment

Each subject filled out the RDC/TMD Axis II questionnaire before and after the end of the treatment. Unfinished questionnaires were excluded from the study. Among the RDC/TMD, characteristic pain intensity, disability points, chronic pain grade, depression index, and grade of nonspecific physical symptoms were analyzed [6].

1. Characteristic pain intensity (CPI)
  The characteristic pain intensity was measured by taking the mean of Q7, Q8, and Q9 and multiplied by 10. The Q7, Q8, and Q9 scores were under the GCP scale.
2. Disability points (DP)
  The disability points were gained by adding points for disability days and disability score. The disability days were obtained by the answer to Q10, and appropriate disability points (0 to 3) were assigned to range of days. For the disability score, the mean value of Q11, Q12, and Q13 was multiplied by 10. Appropriate disability points (0 to 3) were assigned to range of scores. All obtained scores were under the GCP scale.
3. Chronic pain grade (CPG)
  The chronic pain grade classification was termed as following and using previously calculated CPI and DP (Table 2).

**Table 2:** Graded pain score analysis

Degree	Group	Pre-treatment (*n*)
No disability	Grade 0	Absence of TMD and pain prior 6 months
Low disability	Grade I	Low intensity; CPI < 50, DP < 3
	Grade II	High intensity; CPI ≥ 50, DP < 3
High disability	Grade III	Moderately limiting; DP 3–4 (CPI unrelated)
	Grade IV	Severely limiting; DP 5–6 (CPI unrelated)
4. Depression index (DI)
  The mean of the numeric values (scored 0 to 4) to RDC/TMD Axis II Q20 (items: b, e, h, i, k, l, m, n, v, y, cc, dd, ee, f, g, q, z, aa, bb, ff) showed the degree of psychological pain.
5. Nonspecific physical symptoms (pain items included)
  The mean of the numeric values (scored 0 to 4) to RDC/TMD Axis II Q20 related to the pain (items: a, c, d, j, o, p, r, s, t, u, w, x) indicated the degree of atypical somatization and physical pain.
6. Nonspecific physical symptoms (pain items excluded)
  The mean of the numeric values (scored 0 to 4) on RDC/TMD Axis II Q20 (items: c, r, s, t, u, w, x) showed the nonspecific physical symptoms, pain items excluded.

### Statistical analyses

Statistical analysis was accomplished using Wilcoxon signed-rank test (SPSS Inc., ver. 19.0, USA) to evaluate the effectiveness of the BTX-A treatment in the subjects on pain intensity, disability days, graded chronic pain scale, depression, nonspecific physical symptoms, and jaw disability. Statistical significance was defined as *p* < 0.05.

## Results

More than half of the participants (85.7 %) had parafunctional oral habits such as bruxism, clenching, and unilateral mastication. TMD subtypes of the patients were categorized as following according to the JSTMJ classifications (2001) [5]: type I (*n* = 4), type II (*n* = 0), type III (*n* = 5), type IV (*n* = 8), and type V (*n* = 4). The patients were followed up for 2.9 ± 2.0 years by a dentist highly experienced in the TMD treatment.

Overall, intramuscular injections of BTX-A into the masseter and temporalis muscles decreased tenderness and pain. In comparison between pre- and post-treatment results, graded pain score, characteristic pain intensity, disability points, chronic pain grade, and grade of nonspecific physical symptoms showed statistically significant differences after the BTX-A injection therapy (*p* < 0.05). Depression index, however, did not show significant changes (Table 3). Changes in graded pain score (GPS) among the different TMD subtype groups show that disability levels of most patients shifted towards lower degrees of disability after the BTX-A treatments. It also suggests that even after most TMD symptoms subside, some disabilities still remain (Table 4).

**Table 3:** RDC/TMD Axis II analysis

RDC score	Pre-treatment		Post-treatment		*p* value
	Mean	S.D.	Mean	S.D.	
VAS	31.95	13.39	25.71	8.29	0.004*
CPI	55.24	23.86	45.40	15.54	0.017*
DP	3.48	2.11	2.62	1.53	0.011*
CPG	2.90	1.18	2.38	1.02	0.026*
DI	1.15	0.84	1.15	0.88	0.59
NSPI	1.29	0.92	1.11	0.83	0.008*
NSPE	1.07	0.95	0.98	0.90	0.026*

*VAS* Visual Analog Scale, *CPI* characteristic pain intensity, *DP* disability points, *CPG* chronic pain grade, *DI* depression index, *NSPI* nonspecific physical symptoms (pain items included), *NSPE* nonspecific physical symptoms (pain items excluded)

*Statistically significant (*p* < 0.05)

**Table 4:** Graded pain score (GPS)

Degree	Group	Pre-treatment (*n*)	Post-treatment (*n*)
No disability	Grade 0	2	2
Low disability	Grade I	3	6
	Grade II	2	1
High disability	Grade III	5	10
	Grade IV	9	2
Total		21	21

## Discussion

The RDC/TMD is broadly accepted among several diagnostic systems that had been proposed to classify often complex and idiopathic TMDs. Developed by Dworkin and LeResche in 1992, this diagnostic system has been used to classify TMD patients with dual-axis assessments: a clinical and radiographic diagnosis based on pathophysiology (Axis I) and evaluation of psychological status and pain-related disability (Axis II) [6]. With its standardization of diagnostic criteria, this system has been validated for both research and clinical use [7].

The botulinum toxin is known to have seven serotypes (labeled A to G) produced by different *Clostridium botulinum* strains, and serotype A is particularly important and the only commercially available form used in clinics for its biologic activity. The botulinum toxin serotype A (BTX-A), a naturally occurring neurotoxic protein, decreases the muscle contractions by selectively impairing the exocytosis of acetylcholine (ACH) at the neuromuscular junction of presynaptic motor nerve ending. Upon injection, this neurotoxin can effectively reduce the local muscle intensity that can last up to 3 to 6 months before forming a new muscular reinnervation and return to its full function, without significant side effects[8]. BTX-A has been used in treating a variety of medical conditions associated with muscle contraction or pain. In treating orofacial pains, botulinum toxin is usually injected to a number of facial and masticatory muscles including the masseter, temporalis, and other muscles that may associate with TMD.

A growing number of dentists are caring their patients with this neurotoxin for esthetic and therapeutic treatments in oral and maxillofacial areas. According to a survey, 16 % of North American dentists are estimated to use BTX-A in their practices [9]. After introduction of commercial brands of BTX-A such as Botox and Dysport, its injection therapy on orofacial muscles became a valuable adjunct in managing the myofascial components of TMD. Supporting literature dates back three decades, with its successes reported in the various therapeutic applications since the 1980s [10].

BTX-A has been used to treat a wide spectrum of medical conditions such as muscular spasm, hypertrophy, and various cosmetic corrections, and its applications are still expanding. In one report, injection of BTX-A into the lateral pterygoid muscles was recommended as the treatment choice for elderly patients with systemic/neurological diseases who experienced habitual dislocation of the TMJ [11]. BTX-A injections are also known to be useful in treating chronic neuropathic pain, possibly with its analgesic effect that is suggested to employ independent action on peripheral nociceptors by regulating release of various endogenous chemicals such as glutamate, calcitonin gene-related peptide, and substance P [12].

Traumatized TMJ could naturally self-heal or use adaptational mechanism without serious complications [13]. When treating TMD, its diagnosis and future treatment plans should be approached from various perspectives because of possible multifactorial nature of the disorder. It is known that TMD patients recurrently report tenderness in the masticatory musculature regions such as in the temporalis and masseter muscles. There are reports on the clinical use of BTX-A to relieve the tenderness and restore functions of the TMJ. In one report, some patients who were treated with BTX-A had benefited from relief of myofascial pain [14].

BTX-A could also provide a control of parafunctional oral habits that involve the masticatory muscles. BTX-A is considered relatively safe since it does not cross the blood-brain barrier with its effects localized in the peripheral sensory nerves [15]. Binder et al. had reported that even chronic headaches were completely or partially improved on the patients who regularly received BTX-A treatments in facial areas [16, 17].

Moreover, psychosocial factors should not be ignored in diagnosing TMD since these factors sometimes influence one’s physical status [18–21]. Myofascial pains quite often come from psychological factors, and patients who suffer from this etiological pain had shown high chronic pain-related disability and depression/somatization scores [21].

Many studies support that BTX-A effectively diminishes the muscle activity by inhibiting triggers of excessive muscular contractions. In comparison between pre- and post-treatment results of this study, graded pain score, characteristic pain intensity, disability points, chronic pain grade, and grade of nonspecific physical symptoms showed statistically significant differences after the BTX-A injection therapy (*p* < 0.05). However, depression index did not show significant changes. This insists that though states of depression were not affected considerably by the injection therapy, it had positive effects on somatization (with or without pain items) and jaw disability of the TMD patients. Some studies suggest that parafunctional oral habits could be the potential risk factors for myofascial pain and disc derangement [22]. A high prevalence of parafunctional oral habits (85.7 %) in our TMD patients is in accordance with findings of other studies. As changes in graded pain scores before and after the treatment show, BTX-A injections have improved disability levels in almost all TMD subgroups (I, 4; III, 5; IV, 8; V, 4). This result suggests that the BTX-A, which has a direct effect of diminishing muscle contractions, may have therapeutic gains even on complex and idiopathic TMD that involves more than the muscular components of the TMJ.

## Conclusions

Even with the positive outcomes shown from the study, there are needs for more studies performed on a larger sample size, with longer follow-up periods, in order to scrutinize and evaluate the full effects of BTX-A injections. Nevertheless, within the limitation of our study, clinical injection of BTX-A in masticatory musculatures of TMD patients can be considered as a useful supportive treatment option for controlling complex TMD and helping its associated symptoms.

## References

[1] Fricton JR, Kroening RJ, Hathaway K (1988). TMJ and craniofacial pain: diagnosis and management.

[2] Guarda-Nardini L, Manfredini D, Salamone M (2008). Efficacy of botulinum toxin in treating myofascial pain in bruxers: a controlled placebo pilot study. Cranio.

[3] Bjornland T, Gjaerum AA, Moystad O (2007). Osteoarthritis of the temporomandibular joint: an evaluation of the effects and complications of corticosteroid injections compared with injection with sodium hyaluronate. J Oral Rehabil.

[4] Chung JW, Chung SC (2002) Research Diagnostic Criteria for Temporomandibular Disorders RDC/TMD. www.rdc-tmdinternational.org.

[5] Shibuya Y, Takeuchi J, Ikehata N (2007). A clinical study of temporomandibular joint disorders—an analysis based on the Japanese subtype classification. Kobe J Med Sci.

[6] Dworkin SF, LeResche L (1992). Research diagnostic criteria for temporomandibular disorders: review criteria, examinations and specifications, critique. J Craniomandib Disord.

[7] Yap AUJ, Dworkin SF, Chua EK (2003). Prevalence of temporomandibular disorder subtypes, psychologic distress, and psychosocial dysfunction in Asian patients. J Orofac Pain.

[8] Meunier FA, Schiavo G, Molgo J (2002). Botulinum neurotoxins: from paralysis to recovery of functional neuromuscular transmission. J Physiol Paris.

[9] The wealthy dentist, survey (2009) http://thewealthydentist.com/SurveyResults/168-Targeted-Dental-Marketing.htm 2009.

[10] Ludlow CL, Hallett M, Rhew K (1992). Therapeutic use of botulinum toxin. N Engl J Med.

[11] Fu KY, Chen HM, Sun ZP, Zhang ZK, Ma XC (2010). Long-term efficacy of botulinum toxin type A for the treatment of habitual dislocation of the temporomandibular joint. Brit J Oral Maxillofac Surg.

[12] Ranoux D, Attal N, Morain F, Bouhassira D (2008). Botulinum toxin type A induces direct analgesic effects in chronic neuropathic pain. Ann Neurol.

[13] Thiele RB, Marcoot RM (1985). Functional therapy for fractures of the condyloid process in adults. J Oral Maxillofac Surg.

[14] Sidebottom AJ, Patel AA, Amin J (2013). Botulinum injection for the management of myofascial pain in the masticatory muscles. A prospective outcome study. Br J Oral Maxillofac Surg.

[15] Dressler D, Adib Saberi F (2005). Botulinum toxin: mechanisms of action. Eur Neurol.

[16] Binder WJ, Brin MF, Blitzer A, Pogoda JM (2001). Botulinum toxin type A (BOTOX) for treatment of migraine. Semin Cutan Med Surg.

[17] Binder WJ, Brin MF, Blitzer A, Schoenrock LD, Pogoda JM (2000). Botulinum toxin type A (Botox) for treatment of migraine headaches: an open-label study. Otolaryngol Head Neck Surg.

[18] Gale EN, Dixon DC (1989). A simplified psychologic questionnaire as a treatment planning aid for patients with temporomandibular joint disorders. J Prosthet Dent.

[19] Suvinen TI, Reade PC (1995). Temporomandibular disorders: a critical review of the nature of pain and its assessment. J Orofac Pain.

[20] Cimino R, Michelotti A, Stradi R, Farinaro C (1998). Comparison of clinical and psychologic features of fibromyalgia and masticatory myofascial pain. J Orofac Pain.

[21] Rollman GB, Gillespie JM (2000). The role of psychosocial factors in temporomandibular disorders. Curr Rev Pain.

[22] Michelotti A, Cioffi I, Festa P, Scala G, Farella M (2010). Oral parafunctions as risk factors for diagnostic TMD subgroups. J Oral Reahbil.

